# Medication Management of Anxiety and Depression by Primary Care Pediatrics Providers: A Retrospective Electronic Health Record Study

**DOI:** 10.3389/fped.2022.794722

**Published:** 2022-03-17

**Authors:** Talia R. Lester, Yair Bannett, Rebecca M. Gardner, Heidi M. Feldman, Lynne C. Huffman

**Affiliations:** ^1^Division of Developmental Behavioral Pediatrics, Department of Pediatrics, Stanford University School of Medicine, Palo Alto, CA, United States; ^2^Quantitative Science Unit, Department of Medicine, Stanford School of Medicine, Palo Alto, CA, United States

**Keywords:** pediatric mental health, pediatric anxiety, pediatric depression, primary care pediatrics, electronic health records, health services research, developmental and behavioral pediatrics

## Abstract

**Objectives:**

To describe medication management of children diagnosed with anxiety and/or depression by primary care providers within a primary care network.

**Study Design/Methods:**

We performed a retrospective cross-sectional analysis of electronic health record (EHR) structured data from all children seen at least twice in a 4-year observation period within a network of primary care clinics in Northern California. For children who had visit diagnoses of anxiety, depression, anxiety+depression or symptoms characteristic of these conditions, we analyzed the rates and types of medications prescribed. A logistic regression model considered patient variables for the combined sample.

**Results:**

Of all patients 6–18 years old (*N* = 59,484), 4.4% (*n* = 2,635) had a diagnosis of anxiety only, 2.4% (*n* = 1,433) depression only, and 1.2% (*n* = 737) both anxiety and depression (anxiety + depression); 18% of children with anxiety and/or depression had comorbid ADHD. A total of 15.0% with anxiety only (*n* = 357), 20.5% with depression only (*n* = 285), and 47.4% with anxiety+depression (n=343) were prescribed a psychoactive non-stimulant medication. For anxiety and depression only, the top three medications prescribed were sertraline, fluoxetine, and citalopram. For anxiety + depression, the top three medications prescribed were citalopram, sertraline, and escitalopram. Frequently prescribed medications also included benzodiazepines. Logistic regression modeling showed that the depression only and anxety + depression categories had increased likelihood of medication prescription. Older age and mental health comorbidities were independently associated with increased likelihood of medication prescription.

**Conclusions:**

In this network, ~8% of children carried a diagnosis of anxiety and/or depression. Medication choices generally aligned with current recommendations with the exception of use of benzodiazepines.

## Background

According to recent estimates, one in six children aged 6–17 have a treatable mental health disorder (1, 2). Among mental health disorders, depression and anxiety are common, and occur in 3 and 7% of pediatric patients, respectively, in the United States (3). The burden of anxiety and depression in the pediatric population has implications both during childhood and into adulthood (4). In childhood, anxiety disorders and depression are both associated with lower levels of academic achievement, social problems, and disruption in the home (5–7). Both anxiety and depression diagnoses are associated with increased risk of suicide, which is the second most common cause of death in children 10–19 years old (8). In adulthood, anxiety and depression can lead to decreased work productivity, relationship problems, and overall decreased quality of life. Mental health conditions also represent a significant financial burden on the US economy (9, 10). Evidence suggests that early and appropriate treatment of mental health disorders leads to better outcomes than non-treatment (11, 12). Studies suggest that many children with mental health conditions are not receiving adequate care (13). Increasing the use of evidence-based treatment of pediatric mental health conditions would be highly beneficial for both individuals and society.

Most children with anxiety and depression present first to a primary care provider (PCP) (2). Standard of care for childhood anxiety and depression is a multimodal approach, including pharmacological and non-pharmacological treatments. Current guidelines indicate that medication should be considered in moderate and severe cases (14–17). When medication is indicated for pediatric patients with anxiety or depression, the selective serotonin reuptake inhibitor (SSRI) class of medication has a substantial evidence base (14, 18, 19). Among the SSRIs, fluoxetine has the most evidence for good response for both anxiety and depression (18). For anxiety disorders, sertraline and fluvoxamine also have high-quality multisite studies demonstrating efficacy and safety in children (18, 19). For major depressive disorder, fluoxetine is FDA-approved for children, and fluoxetine and escitalopram both are approved for adolescents.

Pediatric patients may be prescribed psychoactive medications that lack evidence for use in the pediatric population (20). Benzodiazepines, once first-line medications for anxiety, are no longer considered first choice medications for general anxiety or panic disorder as a subtype of anxiety (15, 19). Benzodiazepine prescription for pediatric patients is decreasing over time, but they continue to be prescribed without approved indications (21). For example, Toce et al. found that only 24–33% of benzodiazepines prescriptions had an approved indication in adolescents and young adults (21). Knowledge about rates and types of psychotropic medication prescription would be valuable information to guide medical school education and pediatric training about pediatric mental health. Moreover, information about rates and types of medication prescription could serve as baseline information for quality improvement efforts to increase clinician engagement in evidence-based treatment.

A substantial barrier to the provision of adequate pediatric mental health care is the limited number of appropriately trained subspecialists, such as child psychiatrists and developmental-behavioral pediatricians (7, 13). Anderson et al. found that PCPs are the sole managers of 1/3 of children with mental health conditions (22). Given the high prevalence of mental health conditions, the engagement of primary care clinicians in treatment is of paramount importance. However, primary care clinicians frequently report inadequate training about mental health conditions and many remain reluctant to prescribe evidence-based treatments even when they either make the diagnosis or learn that the child was diagnosed by another subspecialty clinician (23–25). Prior studies investigating the role of primary care providers in managing patients with anxiety and depression have utilized provider report to assess comfort level and prescribing practices. Stein et al. surveyed pediatricians and found that 80% agreed that pediatricians should be able to identify conditions of attention-deficit/hyperactivity disorder, eating disorders, substance use, and behavior problems. Eighty-eight percentage agreed that pediatricians should be identifying depression, but only 25% felt pediatricians should be responsible for treating and managing depression (26). Response percentages were similar for pediatric anxiety. Another recent study found that 27% of PCPs do not prescribe psychiatric medications, and of the ones who do, 37% do not feel comfortable prescribing (27).

Surprisingly, few studies include objective data on the clinical care provided for children with anxiety and depression by PCPs (28). Therefore, information about what primary care clinicians actually do in practice (i.e., provider behavior) when caring for children with anxiety and depression, including rates of prescribing medication and types of medications used, is limited. The majority of existing studies include claims or survey data. Limitations of claims data include: incomplete, inaccurate, or missing data, and difficulty tracking specific providers (29, 30). One study of claims data on privately and commercially insured children showed that clinicians do not follow practice guidelines for recommended duration of treatment with antidepressant medications (31).

In this study, we used objective electronic health record data to describe the rates of medication management for children with anxiety and/or depression by pediatric PCPs and the types of medications prescribed in a large primary care network. We anticipated that even when primary care clinicians include depression or anxiety as a visit diagnosis, the rates of prescribing psychoactive medications would be low. We identified patient characteristics associated with prescription of medication to understand when primary care clinicians were willing to prescribe medication. We hypothesized that older age and higher number of comorbidities would be associated with likelihood to be prescribed medication for anxiety or depression. We conceptualize these data as providing a foundation for improving pediatric training and for conducting quality improvement activities in pediatric mental health.

## Methods

### Setting and Population

Packard Children's Healthcare Alliance (PCHA) is a community-based network of 25 primary care offices in Northern California, affiliated with Lucille Packard Children's Hospital and Stanford Children's Health. The Stanford University School of Medicine institutional review board (IRB) determined that this study was not human subjects research.

### Study Design

We conducted a retrospective review of electronic health records for a cohort including all pediatric patients 6–18 years old seen at PCHA clinics for at least two visits during a 4-year study period (October 1, 2015 to September 30, 2019). Two visits were required within the study period in order to ensure that included patients were active within the network.

### Data Sources

**Figure 1:**
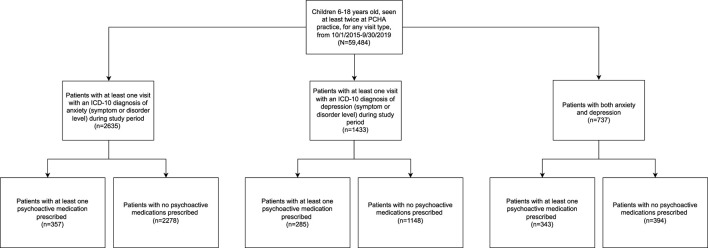
Cohort selection.

Deidentified structured data from all office encounters were included. Data from all practices were combined. Figure 1 shows the study cohort flow-chart. Patient data included age in years, sex, race, ethnicity, and medical insurance. Children under 6 years old were not included due to low rate of medication prescription (Supplementary Material 1).

### Measures

#### Identifying Patients With Anxiety and Depression

Patients with anxiety and/or depression were identified based on the presence of at least one ICD-10 visit diagnosis code associated with anxiety or depression. Patients with these diagnoses were included, irrespective of pre-existing diagnoses. Both disorder-level codes (“F codes”, for example: “Generalized Anxiety Disorder”) and symptom-level codes (“R codes”, for example: “worries”) were included. See Supplementary Material 2 for a full list of ICD-10 codes used. Once selected for inclusion, patients were defined as an “anxiety patient” if they had only an anxiety diagnosis (with no depression), “depression patient” if they had only a depression diagnosis (with no anxiety) or “anxiety+depression patient” if they had both anxiety and depression diagnoses during the study period, although the two diagnoses were not required to be present at the same visit.

#### Defining Medication List

A list of all medications prescribed during the study period at visits with anxiety and/or depression diagnoses was reviewed, and a comprehensive list of psychoactive medications (excluding stimulants) was generated. Patients were defined as being prescribed medication if they had a prescription for any medication from this list associated with the encounter, including both initial prescriptions and refill prescriptions.

#### Defining Comorbidities

Behavioral and mental health comorbidities were identified based upon the presence of ICD-10 diagnosis codes associated with the following: Attention-deficit/hyperactivity disorder (ADHD), Sleep Disorders, Trauma and Stressor Related Disorders, Autism Spectrum Disorders (ASD), Obsessive Compulsive and Related Disorders, Feeding and Eating Disorders, Disruptive, Impulse Control, and Conduct Disorders, Substance-Related and Addictive Disorders, and Bipolar and Related Disorders. These disorders were chosen based on the Diagnostic and Statistical Manual of Mental Disorders, 5th Edition (DSM-5) (32). The comorbid diagnosis was not required to be present at the same visit as the anxiety and/or depression diagnosis.

### Statistical Analyses

#### Descriptive Statistics

For patients with anxiety and/or depression, frequency counts, proportions, means and standard deviations were used to summarize data representing patient characteristics (demographics, comorbidities) and most commonly prescribed medications.

#### Regression Analysis

A logistic regression model including patients with anxiety, depression, and anxiety + depression was performed to determine association of patient characteristics with likelihood to be prescribed medication. Independent variables included: age group (6–12 and 13–18 years old), sex, insurance (private, public, military, or unknown), referral to developmental-behavioral pediatrics or psychiatry services within Stanford Children's Health, number of mental health and/or behavioral comorbidities (0, 1, or 2+), and primary diagnostic category (anxiety, depression, or anxiety + depression). Anxiety was selected as the reference group because it is the largest group and is the most prevalent condition across the included age range. We calculated adjusted odds ratios (aOR) and 95% confidence intervals (CI) from the regression model.

Since there was a high rate of missing race and ethnicity data in our cohort (~30%), race and ethnicity were not included in the regression model. Imputation was not attempted since missingness was highly dependent on practice/clinic.

Data cleaning and reformatting were performed using R version 3.6.2. Analyses were conducted using SPSS 26 and 27 and R Version 3.6.2 (33, 34). All statistical tests were two-sided and conducted at the 0.05 significance level.

## Results

### Patient Characteristics and Prevalence of Anxiety and/or Depression

Of 59,484 patients 6–18 years old seen at least twice during the study period for any reason, 4,498 (7.5%) had a diagnosis of anxiety and/or depression.

Of the 4,498, 41.2% were male, 40.6% were white, and 75.7% were privately insured. Table 1 describes characteristics of the 4,498 patients: 2,385 (53.0%) had at least one visit with a diagnosis of anxiety only (symptom or disorder level), *n* = 1,390 (30.9) had at least one visit with a diagnosis of depression only (symptom or disorder), and *n* = 723 (16.1%) had diagnoses of both anxiety and depression (anxiety + depression) (symptom or disorder).

**Table 1:** Characteristics of patients with anxiety and depression diagnoses (*N* = 4,498).

**Patient characteristics**	***n* (%)**
**Age group at first anxiety and/or depression visit, years**	
6–12	1,672 (37.2)
13–18	2,826 (62.8)
Mean age	13.6 (SD = 3.2)
Male	1,851 (41.2)
Female	2,647 (58.8)
**Race**	
Asian	434 (9.6)
Black or African American	147 (3.3)
White or Caucasian	1,824 (40.6)
Other	832 (18.5)
Unknown	1,261 (28.0)
**Ethnicity**	
Hispanic	513 (11.4)
Not Hispanic	2,533 (56.3)
Unknown	1,452 (32.3)
**Insurance**	
Private	3,403 (75.7)
Public	927 (20.6)
Military	126 (2.8)
Unknown	42 (0.9)
**Diagnosis categories**	
Anxiety only (anxiety)	2,385 (53.0)
Depression only (depression)	1,390 (30.9)
Both anxiety and depression during study period (anxiety + depression)	723 (16.1)
**Comorbidities in patients with anxiety and depression**	
Attention-deficit/hyperactivity disorder	832 (18.7)
Sleep disorders	462 (10.3)
Trauma and stressor related disorders	213 (4.7)
Autism spectrum disorder	153 (3.4)
Obsessive compulsive and related disorders	123 (2.7)
Feeding and eating disorders	120 (2.7)
Disruptive, impulsive control, and conduct disorders	84 (1.9)
Substance-related and addictive disorders	63 (1.4)
Bipolar and related disorders	29 (0.6)

*SD, standard deviation*.

#### Comorbid Behavioral and Mental Health Conditions in Patients With Anxiety and/or Depression

Table 1 also includes the most common comorbid behavioral and mental health disorders: Attention-deficit/hyperactivity disorder (18.7%), sleep disorders (10.3%), trauma and stressor-related disorders (4.7%) and autism spectrum disorder (3.4%). Eight hundred and fifteen (34.2%) of patients with anxiety, 466 (33.5%) of patients with depression, and 357 (49.4%) of patients with anxiety + depression, had one or more co-existing behavioral or mental health condition.

#### Medications for Patients With Anxiety and/or Depression

Of 2,385 patients with anxiety, 15.0% (*n* = 357) were prescribed a psychoactive (non-stimulant) medication. Of 1,390 children with depression, 20.5% (*n* = 285) were prescribed a psychoactive medication. Of 723 children with anxiety + depression diagnoses during the study period, 47.4% (*n* = 343) were prescribed a psychoactive medication.

Table 2 shows the ten most commonly prescribed medications for each diagnosis. Most medications were in the class of serotonin reuptake inhibitors (SSRIs). For patients with anxiety, the top three prescribed medications were: (1) sertraline (prescribed to 52% of all anxiety patients who were prescribed medication during the study period) and (2) citalopram (28%) and fluoxetine (28%). For patients with depression, the top three prescribed medications were: (1) fluoxetine (50%), (2) citalopram (43%), and (3) sertraline (40%). For patients with anxiety + depression, the top three prescribed medications were: (1) citalopram (48%), (2) sertraline (41%), and (3) escitalopram (32%).

**Table 2:** Most commonly used medications by diagnosis category.

**Anxiety only**			**Depression only**			**Anxiety and depression**		
**Medication name**	**Number of patients**	**Percentage of patients prescribed medication (total = 357)**	**Medication name**	**Number of patients**	**Percentage of patients prescribed medication (total = 285)**	**Medication name**	**Number of patients**	**Percentage of patients prescribed medication (total = 343)**
Sertraline	184	52	Fluoxetine	144	50	Citalopram	166	48
Fluoxetine	99	28	Citalopram	121	43	Sertraline	140	41
Citalopram	99	28	Sertraline	115	40	Escitalopram	110	32
Escitalopram	83	23	Escitalopram	93	33	Fluoxetine	110	32
Guanfacine	67	19	Aripiprazole	45	16	Aripiprazole	41	12
Lorazepam	59	16	Lamotrigine	37	13	Lorazepam	36	10
Clonidine	34	9	Guanfacine	26	9	Duloxetine	24	7
Atomoxetine	28	7	Trazodone	20	7	Guanfacine	24	7
Alprazolam	25	7	Venlafaxine	20	7	Lamotrigine	22	6
Aripiprazole	24	7	Risperidone	18	6	Gabapentin	16	5

Two benzodiazepines appear on the list of ten most commonly prescribed medications for anxiety. Lorazepam was prescribed to 16.0% of patients and alprazolam was prescribed to 7.0% of patients. Lorazepam also appears on the list of ten most commonly prescribed medications for anxiety + depression (prescribed to 10% of patients). Benzodiazepines were not on the list of ten most commonly prescribed medications for depression.

#### Diagnoses Associated With Benzodiazepine Prescription

We collected the diagnostic codes most frequently associated with benzodiazepine prescription for patients with anxiety, depression, and anxiety + depression as a combined category. F41.9 (anxiety disorder), F32.9 (major depressive disorder), and F41.0 (panic disorder) were the top three anxiety and/or depression diagnostic codes associated with prescription. Z00.129 (encounter for routine child health examination), J02.9 (acute pharyngitis), and Z23 (encounter for immunizations) were the top three non-anxiety/depression diagnostic codes associated with benzodiazepine prescription.

#### Medications for Patients With Symptom-Level Diagnoses

For patients with anxiety, none of the 51 patients with symptom-level diagnoses only, such as “worries”, received medication. For patients with depression, only 1 of the 216 patients (<1.0%) with symptom-level diagnoses only, such as “sadness”, received medication. For patients with anxiety + depression, 0 of the 2 patients with symptom-level anxiety and depression received medication.

### Predictors of Medication Prescription: Regression Model

A logistic regression model was performed to determine patient factors associated with likelihood to be prescribed medication for patients with anxiety and/or depression (Table 3). See Supplementary Material 3 for proportions of patients in each diagnostic category (i.e., anxiety, depression, anxiety + depression) who were prescribed medication. The model explained 15% of the variance (Nagelkerke *R*^2^) in medication prescription. Compared to 6–12 year old children, 13–18 year old children had 2.12 higher odds of being prescribed medication (95% CI: 1.78–2.53, *p* < 0.001). Compared to children with anxiety, children with depression had 1.26 higher odds of being prescribed medication (95% CI: 1.05–2.50, *p* = 0.01) and children with both anxiety and depression had 4.02 higher odds of being prescribed medication (95% CI: 3.31–4.88, *p* < 0.001). Compared to children without comorbidities, children with behavioral and mental health comorbidities were more likely to be prescribed medication (one comorbidity: aOR 1.78, 95% CI: 1.50–2.10, *p* < 0.001), (two or more comorbidities: aOR 2.94 95% CI: 2.30–3.75, *p* < 0.001).

**Table 3:** Patient characteristics associated with medication prescription for patients with anxiety only, depression only, and both (anxiety/depression).

**Patient characteristics**	**Number of patients (*n*)** **(*N* = 4,498)**	**Medication odds ratio (95% CI)**	***P*-value**
**Age**			
6–12	1,672	Ref	
13–18	**2,826**	**2.12 (1.78–2.53)**	**<0.001**
**Sex**			
Female	2,647	0.89 (0.77–1.04)	0.15
**Insurance**			
Private	3,403	Ref	
Public	927	0.85 (0.70–1.03)	0.10
Military	126	1.09 (0.70–1.70)	0.71
NA	42	0.86 (0.37–1.97)	0.72
**Primary diagnosis category**			
Anxiety	2,385	Ref	
Depression	**1,390**	**1.26 (1.05–1.50)**	**0.01**
Anxiety + Depression	**723**	**4.02 (3.31–4.88)**	**<0.001**
**Number of comorbidities**			
0	2,860	Ref	
1	**1,250**	**1.78 (1.50–2.10)**	**<0.001**
2+	**388**	**2.94 (2.30–3.75)**	**<0.001**
Internal Referral to DBP or Psychiatry	583	1.08 (0.86–1.34)	0.52

*Note: Overall model Nagelkerke R-squared = 0.15*.

Patient sex, insurance type (private, public, military, or none/unknown), and referral to developmental-behavioral pediatrics and/or psychiatry within Stanford Children's Health were not associated with the likelihood of being prescribed medication.

## Discussion

We found that, within this population of children in Northern CA, 7.5% of patients had a diagnosis of anxiety and/or depression recorded in their primary care electronic health record. Comorbid developmental, behavioral, and mental health conditions were common in all three subcohorts based on diagnostic group. 34.2% of patients with anxiety, 33.5% of patients with depression, and 49.4% of patients with anxiety + depression had one or more co-existing behavioral or mental health conditions. Psychoactive medications were prescribed to 15.0% of patients with anxiety, 20.5% with depression, and 47.4% of patients with anxiety + depression. This aligns with both prior claims data studies as well as provider survey studies which indicate that primary care providers play a major role in medication management for children with anxiety and depression (26, 35). We also found that patients very rarely received medication without a disorder-level ICD-10 code. This suggest that clinician behavior aligns with guideline recommendations that medication is not indicated in most mild cases of anxiety and depression (14, 15).

Current literature indicates that SSRIs have the most evidence of beneficial effect in pharmacological treatment of anxiety and depression in pediatric patients. Clinical practice guidelines and practice parameters also support the use SSRI medications; fluoxetine and escitalopram recommended as first line for depression (19). High quality, NIH-sponsored multisite trials have supported safety and efficacy of fluoxetine (19). In our study, patients with anxiety or with depression most commonly were prescribed sertraline, citalopram, and fluoxetine. For patients with anxiety+depression, citalopram, sertraline, and escitalopram most commonly were prescribed. These findings indicate that the medications most commonly used by PCPs for anxiety and depression generally align with current evidence.

We were surprised to find that a substantial proportion of patients who were prescribed medication during the study period were prescribed benzodiazepine medications at some point. Lorazepam appears on the list of most commonly prescribed medications for patients with anxiety only (16% of patients prescribed medication) and anxiety and depression (10% of patients prescribed medication). This finding aligns with other recent literature suggesting that benzodiazepines are commonly prescribed to pediatric patients despite limited evidence for beneficial effect in pediatric patients (18, 20, 36). There are also concerns about safety and the potential of diversion (37, 38). We wondered if benzodiazepines may be prescribed to children with anxiety and depression for other reasons, such as seizure disorders or procedural anxiolysis. We investigated the most common non-anxiety/depression related diagnostic codes associated with benzodiazepine prescription in patients with anxiety, depression, and anxiety + depression, and we found that they did not correlate with a clear non-psychiatric indication that would explain the use of benzodiazepines. Of note, panic disorder (which can be an indication for benzodiazepine use in some clinical scenarios) appears in the most common anxiety- and depression-related diagnostic codes. Further study is needed to understand the rationale for use of these medications by PCPs in this network. In particular, it would be important to determine if subspecialist involvement was associated with prescription of benzodiazepines in these pediatric patients.

We investigated patient factors associated with likelihood to be prescribed medication for patients with anxiety, depression, and anxiety + depression. The model accounted for 15% of the variance in medication use. Thus, it appears that other factors not assessed here are important to the decision to treat with medication. We also found that patients with depression alone and patients with anxiety + depression were more likely to be prescribed medication than patients with anxiety alone. Older age and higher number of comorbidities were associated with increased likelihood to be prescribed medication. Our findings align with a prior interview-based study, which have found that pediatricians are less likely to prescribe medication for anxiety than for depression, due to clinician perception that anxiety disorders are less impairing than depression (39). We speculate that primary care clinicians may be more comfortable identifying anxiety and depression as clear targets for treatment in older patients. We also wonder if the population of patients with both anxiety and depression represents a more clinically severe and/or complex population, where clinicians may perceive that medication is more justifiable. Further research in this area is warranted.

This study has several limitations. First, we were not able to consider the effects of referral to external psychiatry resources or referral to psychotherapy as this information was not captured in the electronic health record structured data. Second, a more thorough understanding of PCP recommendation of non-pharmacologic treatments to these children will require chart review. Although the primary care network from which our data was obtained is community-based, the patient demographics are not representative of demographics in California (our data set includes higher percentage white and private insurance), limiting the generalizability of the findings (40). Data from all practices was combined in the analysis. For that reason, we would not be able to discern prescribing differences at the practice level.

## Conclusions

This study of a large community-based network of pediatrics practices showed that medications prescribed by primary care providers for anxiety and depression generally align with current recommendations. The most commonly used medications were SSRIs. Further study is needed to understand why providers use benzodiazepines, which have less evidence for beneficial effect, and more concern for side effects, diversion, and unintended recreational use. PCPs prescribe medications more frequently for patients with anxiety + depression than for patients with either diagnosis alone. Older age and higher number of comorbidities were associated with increased likelihood to be prescribed medication. Having depression only or anxiety + depression were both associated with increased likelihood to be prescribed medication relative to anxiety alone. Further study also is needed to investigate how PCPs collaborate with subspecialists in the care of patients with anxiety and depression.

## Data Availability Statement

The data analyzed in this study is subject to the following licenses/restrictions: available with Stanford IRB and privacy office approval. Requests to access these datasets should be directed to huffmanl@stanford.edu and ybannett@stanford.edu.

## Author Contributions

TL, YB, RG, HF, and LH contributed to conception and design of the study. RG completed initial data cleaning and organization. TL, RG, YB, and LH performed statistical analysis. TL wrote the first draft the manuscript. YB, RG, HF, and LH contributed to manuscript revision and read, and approved the submitted version.

## Funding

Partial funding was provided by Developmental-Behavioral Pediatrics Fellowship Training from the Maternal Child Health Bureau of Health Resources and Services Administration T77MC09796.

## Conflict of Interest

The authors declare that the research was conducted in the absence of any commercial or financial relationships that could be construed as a potential conflict of interest.

## Publisher's Note

All claims expressed in this article are solely those of the authors and do not necessarily represent those of their affiliated organizations, or those of the publisher, the editors and the reviewers. Any product that may be evaluated in this article, or claim that may be made by its manufacturer, is not guaranteed or endorsed by the publisher.
