# Supplementary material for: Medication Management of Anxiety and Depression by Primary Care Pediatrics Providers: A Retrospective Electronic Health Record Study
Source: Front Pediatr. 2022 Mar 17;10:794722. doi: 10.3389/fped.2022.794722 (PMC8970594; doi:10.3389/fped.2022.794722)
Supplement: Supplementary file 1 [file Data_Sheet_1.PDF]

Supplement 1: Medication Prevalence by Age

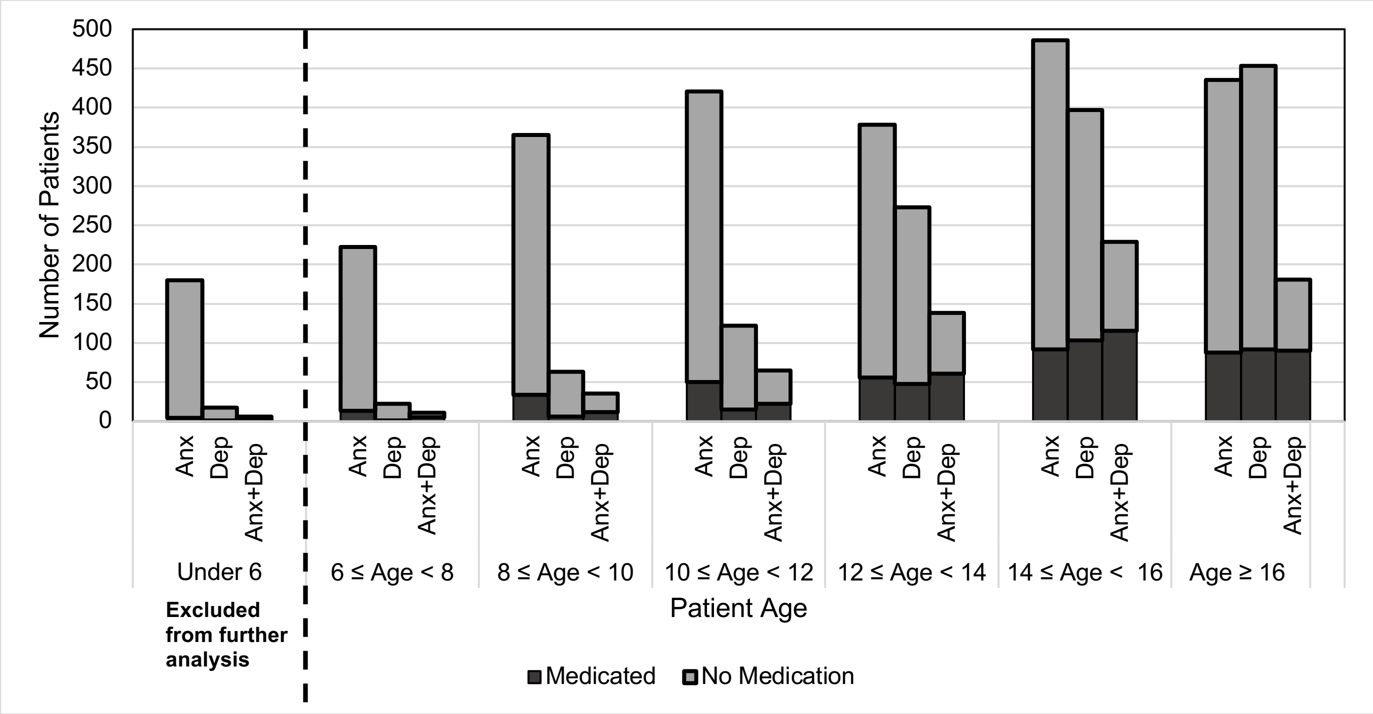

Supplement 2 : ICD 10 Codes and Text descriptors Used for Study Outcomes

Condition	ICD-10 Diagnosis Code	ICD-10 Text Descriptors
Anxiety		
Symptom Level	R45.2	Unhappiness (worries NOS)
	R45.82	Worries
	R45.89	Feeling worried, anxious appearance, feeling anxious
Disorder Level	F06.4,F10.180,F10.280,F12.980,F13.180,F13.280,F13.980,F14.180,F14.280,F14.980,F15.180,F15.280,F15.980,F16.180,F16.280,F16.980,F18.180,F18.280,F18.980,F19.180,F19.280,F19.980,F31.3,F40.0,F40.00,F40.01,F40.02,F40.10,F40.11,F40.210,F40.218,F40.220,F40.228,F40.23,F40.230,F40.231,F40.233,F40.240,F40.241,F40.242,F40.243,F40.248,F40.291,F40.298,F40.8,F40.9,F41.0,F41.1,F41.3,F41.8,F41.9,F93.0,F94.0	All
Depression		
Symptom Level	R45.7	State of emotional stress or shock
	R45.81	Low self esteem
	R45.84	Anhedonia
	R45.851	Suicidal ideations
	R45.86	Emotional lability
	R45.89	Sad, sad mood, feeling sad, sadness, feeling of sadness, at risk for self harm, depressed affect, difficulty coping, dysphoric mood, feelings of worthlessness, feels depressed, flat affect, non-suicidal depressed mood, non-suicidal self harm, self-esteem disturbance, suicidal risk, tearfulness, thoughts of self harm
Disorder Level	F06.30,F06.31,F06.32,F06.34,F10.14,F10.24,F10.94,F11.14,F11.24,F11.94,F13.14,F13.24,F13.94,F14.14,F14.24,F14.94,F15.14,F15.24,F15.94,F16.14,F16.24,F16.94,F18.14,F18.24,F18.94,F19.14,F19.24,F19.94,F32.0,F32.1,F32.2,F32.3,F32.4,F32.5,F32.8,F32.81,F32.89,F32.9,F33.0,F33.1,F33.2,F33.3,F33.40,F33.41,F33.42,F33.8,F33.9,F34.1,F34.81,F34.89,F34.9	All

Supplement 3: Medication Prescription by Patient Factors for 6-18 year old patients

	Anxiety (N=2385)		Depression (N=1390)		Anxiety+Depression (N=723)	
	Medicated	Nonmedicated	Medicated	Nonmedicated	Medicated	Nonmedicated
Age						
6-12	125/1185 (10.5%)	1060/1185 (89.5%)	40/325 (12.3%)	285/325 (87.7%)	61/162 (37.7%)	101/162 (62.4%)
3-18	232/1200 (19.3%)	968/1200 (80.7%)	245/1065 (23.0%)	820/1065 (77.0%)	282/561 (50.3%)	279/561 (49.7%)
Sex						
Female	184/1352 (13.6%)	1168/1352 (86.4%)	166/833 (19.9%)	667/833 (80.1%)	218/462 (47.2%)	244/462 (52.8%)
Male	173/1033 (16.7%)	860/1033 (83.3%)	119/557 (21.4%)	438/557 (78.6%)	125/261 (47.9%)	136/261 (52.1%)
Insurance						
Private	280/1869 (15.0%)	1589/1869 (85.0%)	212/1004 (21.1%)	792/1004 (78.9%)	263/530 (49.6%)	267/530 (50.4%)
Public	69/425 (16.2%)	356/425 (83.8%)	59/343 (17.2%)	284/343 (82.8%)	64/159 (40.3%)	95/159 (59.8%)
Military	6/65 (9.2%)	59/65 (90.8%)	11/33 (33.3%)	22/33 (66.7%)	13/28 (46.4%)	15/28 (53.6%)
NA	2/26 (7.7%)	24/26 (92.3%)	3/10 (30.0%)	7/10 (70.0%)	3/6 (50.0%)	3/6 (50.0%)
Number of Co-morbidities						
0	170/1570 (10.8%)	1400/1570 (89.2%)	160/924 (17.3%)	764/924 (82.7%)	152/366 (41.5%)	214/366 (58.5%)
1	133/637 (20.9%)	504/637 (79.1%)	92/369 (24.9%)	277/369 (75.1%)	123/244 (50.4%)	121/244 (49.6%)
2+	54/178 (30.3%)	124/178 (69.7%)	33/97 (34.0%)	64/97 (66.0%)	68/113 (60.2%)	45/113 (39.8%)
Comorbid Diagnoses						
ADHD	116/452 (25.7%)	336/452 (74.3%)	69/220 (31.4%)	151/220 (68.6%)	93/170 (54.7%)	77/170 (45.3%)
ASD	40/104 (38.5%)	64/104 (61.5%)	4/30 (13.3%)	26/30 (86.7%)	15/19 (78.9%)	4/19 (21.1%)
Sleep	38/227 (16.7%)	189/227 (83.3%)	27/118 (22.9%)	91/118 (77.1%)	60/117 (51.3%)	57/117 (48.7%)
Trauma and Stressor Related Disorders	10/82 (12.2%)	72/82 (87.8%)	16/74 (21.6%)	58/74 (78.4%)	30/57 (52.6%)	27/57 (47.4%)
Referral to DPB or Psychiatry	43/232 (18.5%)	189/232 (81.5%)	48/208 (23.1%)	160/208 (76.9%)	63/143 (44.1%)	80/143 (56.0%)
